# Evaluation of the association between the *TAS1R2* and *TAS1R3* variants and food intake and nutritional status in children

**DOI:** 10.1590/1678-4685-GMB-2016-0205

**Published:** 2017-05-11

**Authors:** Silvia V. Melo, Grasiela Agnes, Márcia R. Vitolo, Vanessa S. Mattevi, Paula D.B. Campagnolo, Silvana Almeida

**Affiliations:** 1 Laboratório de Biologia Molecular, Universidade Federal de Ciências da Saúde de Porto Alegre, Porto Alegre, RS, Brazil; 2 Programa de Pós-Graduação em Ciências da Saúde, Universidade Federal de Ciências da Saúde de Porto Alegre, Porto Alegre, RS, Brazil; 3 Centro de Ciências da Saúde, Universidade do Vale dos Sinos (UNISINOS), Porto Alegre, RS, Brazil

**Keywords:** food intake, polymorphism, *TAS1R2* gene, *TAS1R3* gene, taste receptors

## Abstract

Taste perception plays a key role in determining individual food preferences and dietary habits and may influence nutritional status. This study aimed to investigate the association of *TAS1R2* (*Ile191Val* - rs35874116) and *TAS1R3* (*-1266 C/T -* rs35744813) variants with food intake and nutritional status in children followed from birth until 7.7 years old. The nutritional status and food intake data of 312 children were collected at three developmental stages (1, 3.9 and 7.7 years old). DNA was extracted from blood samples and the polymorphisms were analyzed by real-time polymerase chain reactions (qPCR) using hydrolysis probes as the detection method. Food intake and nutritional status were compared among individuals with different single nucleotide polymorphism (SNP) genotypes. At 3.9 years old, children homozygous (*Val/Val*) for the *TAS1R2 Ile191Val* polymorphism ingested less sugar and sugar-dense foods than children who were *Ile carriers. This finding demonstrated that a genetic variant of the T1R2 taste receptor is associated with the intake of different amounts of high sugar-content foods in childhood. This association may provide new perspectives for studying dietary patterns and nutritional status in childhood.

## Introduction

The prevalence of childhood obesity throughout the world has increased rapidly in recent decades (Oliveira and Fisberg, 2003; Allison *et al*., 2008; WHO, 2010). The perception of flavors (sweet, bitter, salty, sour and *umami*) can play a key role in determining the preference for foods and dietary habits, thereby influencing nutritional status (Garcia-Bailo *et al*., 2009). Individual food preferences are based on physiological and nutritional factors, as well as economic and socio-cultural aspects. However, the sensory properties of food have a critical effect on dietary choices, and flavor can be a major determinant of food selection (Leterne *et al*., 2008; Garcia-Bailo *et al*., 2009). The T2R family of taste receptors is associated with the perception of bitter taste while the T1R family is associated with the perception of sweet and *umami* tastes (Nelson *et al*., 2001; Zhao *et al*., 2003). The T1R family consists of the T1R1, T1R2 and T1R3 receptors that are coupled to a heterodimeric G protein (Hoon *et al*., 1999; Zhao *et al*., 2003). The T1R receptors combine to form heterodimeric receptor complexes: T1R1-T1R3 is responsive to L-amino acids (*umami* taste perception) and T1R2-T1R3 is responsive to a variety of molecules, including natural sugars and artificial sweeteners (perception of sweetness) (Zhao *et al*., 2003). The receptor complex T1R2-T1R3 is encoded by the *TAS1R2* and *TAS1R3* genes, both of which are located on chromosome 1 (Liao and Schultz, 2003; Fushan *et al*., 2009). The *TAS1R2* gene is characterized by a high natural diversity and the *Ile191Val* polymorphism (rs35874116) leads to the substitution of an isoleucine for a valine at position 191, which lies in a putative ligand-binding domain. Eny *et al*. (2010) reported an association between this polymorphism and sugar intake and differences in the response to dietary counseling. Fushan *et al*. (2009) described two polymorphisms in the *TAS1R3* gene, -1572 C/T (rs307355) and *-1266 C/T* (rs35744813), that are in strong linkage disequilibrium and could be related to the perception of the taste of sucrose; these polymorphisms account for 16% of the variation in sensibility.

Genetic variation in taste receptors may be associated with greater sensitivity to particular flavors and result in a higher intake of some foods considered to be palatable but which are highly energetic and may be associated with an increased risk of overweight. The aim of this study was to examine the association of the *Ile191Val* SNP in the *TAS1R2* gene and the *-1266 C/T* SNP in the *TAS1R3* gene with food intake and nutritional status at three developmental phases in children followed up until 7.7 years old.

## Subjects and Methods

### Subjects

The subject cohort was drawn from a randomized trial conducted in the first year of life to assess the impact of dietary counseling given to mothers during the first year of the infants' lives and dealt with food consumption, nutritional status and lipid profile (Vitolo *et al*., 2010). The initial sample consisted of 500 children born from October 2001 to July 2002. Mothers were recruited from the maternity ward of the only publicly funded city hospital in São Leopoldo, a city in the southern Brazilian state of Rio Grande do Sul. HIV-positive mothers and infants with congenital malformations were excluded from the study. All eligible mothers were informed by field workers about the overall aims of the study and all research procedures. The intervention group received dietary advice during home visits in the first year of life, based on the `Ten Steps to Healthy Feeding', a Brazilian national health policy for primary care, supported by the World Health Organization (Brasil Ministério da Saúde and Organização Pan-Americana da Saúde, 2002). The dietary advice encouraged exclusive breastfeeding during the first six months, after which the mothers were encouraged to continue breastfeeding and gradually introduce foods. Both groups (control and intervention) received visits at six and 12 months and routine follow-up by their pediatricians. This intervention was not the primary objective of the present research and participation in the intervention/control group was considered a confounding factor in subsequent statistical analyses.

The study sample consisted of 312 children who completed the initial phase of the study. The anthropometric measures and dietary data for each child were evaluated at three time points (1, 3.9 and 7.7 years old) at the childs home. The main reason for the loss of subjects was the inability to locate the child's home, usually because the family had moved to another city. Other reasons included refusal to continue in the study and children/maternal death. Race or ethnicity was classified by the interviewer based on skin color. Additional details of the traits studied are described in Vitolo *et al*. (2008).

The study protocol was approved by the ethics committee of the Universidade Federal de Ciências da Saúde de Porto Alegre and was in accordance with The Code of Ethics of the World Medical Association (Declaration of Helsinki) for experiments involving humans. All parents/guardians of the participants provided written informed consent before commencing the study.

### Nutritional status and dietary data

Undergraduate nutrition students were trained to collect data related to the children's anthropometric, dietary, sociodemographic and health status variables. Food intake was assessed through 24 h dietary recalls. At 1 year old, one 24 h dietary recall was recorded for each child. At 3.9 and 7.7 years old, two 24 h dietary recalls were recorded on two nonconsecutive days and the mean values were used in the analyses. The first dietary recall was done at the subjects home and the second was done in the municipal health unit. Portion sizes were confirmed with the aid of an album that was specifically designed for this study and contained photographs of utensils and foods, as well as domestic measurements, such as cups, tablespoons and teaspoons. The Nutrition Support Software (Nutwin 1.5, Federal University of São Paulo, São Paulo, Brazil) was used to convert the average sizes into grams for different measurements and to estimate the energy intake from the selected foods (Philippi, 2002; NEPA, 2006).

The energy intake from foods with a high sugar content (sugar-dense foods – SDF) was calculated from the two dietary recalls. The foods were classified as SDF when the sugar content was ≥50% and included items such as soda, Jell-O, candies and artificial juice. The foods were chosen based on the results of a study conducted in Brazil that indicated which foods were most commonly present in childrens diets. The average food intake from the two dietary recalls was used for the analysis.

At 1 year old, each childs height and weight were evaluated. At ages 3.9 and 7.7 years, the height, weight, tricipital and subscapular skinfold thickness and waist circumference were measured. The body mass index (BMI) was calculated as weight (kg)/height^2^ (m^2^) and the values were transformed into Z-score (BMIzs). Details of the measurement procedure have been described by Vitolo *et al*. (2010). The percent variation in BMI for each child was computed with the formula [(BMI_a_ – BMIb)/BMIb]x100, where a = final BMI and b = initial BMI.

### DNA analyses

At the age of 3.9 years, blood samples were collected for hematological analyses (data not used in this study) and for DNA extraction. The blood samples were collected and transported to the Molecular Biology Laboratory of the Universidade Federal de Ciências da Saúde de Porto Alegre. Genomic DNA was extracted from peripheral blood leukocytes using a standard salting-out procedure (Lahiri and Nurnberger, 1991). The DNA samples are currently stored in this same laboratory. The *Ile191Val* polymorphism of the *TAS1R2* gene and the *-1266 C/T* polymorphism of the *TAS1R3* gene were detected by allelic discrimination with TaqMan 5′-nuclease assays using a Step-One-Plus^®^ instrument (real-time PCR, Applied Biosystems, CA, USA). The reactions were run on 96-well plates with fast thermal cycling conditions and the reagent concentrations were: 1X TaqMan^®^ genotyping master mix, 1X TaqMan^®^ genotyping assays (C_55646_20 and AHCSYY5), 10 ng of DNA and nuclease-free water.

### Statistical analysis

Allele frequencies were estimated by gene counting. A chi-squared goodness-of-fit test was used to determine whether the distribution of observed genotype frequencies agreed with those expected under Hardy-Weinberg equilibrium. The normality of the variables distribution was analyzed with the Kolmogorov-Smirnov test and by examining the histogram. BMI variation from 3.9 to 7.7 years old, SDF and sugar intake variables were ln transformed prior to analysis because of their skewed distribution, but the untransformed values are shown. The data for dietary intakes and parameters of adiposity were compared between genotype groups by using a General Linear Model with type III sum of squares. To construct the models, the daily energy intake, SDF and sugar intake, BMI Z-score and BMI variations were included as dependent variables, the genotype groups were used as fixed factors and intervention or control group status, sex and exact age, and exact age difference between each child's evaluations were included as covariates. The models for examining the association of polymorphisms with average daily energy intake, SDF and sugar intake were adjusted for intervention or control group status, sex and exact age. For the BMI Z-scores, only the variable intervention or control group status was included in the model. For variation in BMI, the variables included in the model were the exact age difference between each child's evaluations, intervention or control group status and sex. All tests and transformations were done using the Statistical Package for Social Sciences, version 16.0 (SPSS, Chicago, IL, USA). Values of p ≤ 0.05 were considered significant.

## Results

The number of children analyzed differed among the polymorphisms in each phase of the study because some biological samples were not analyzed for all polymorphisms. In samples collected from children at 3.9 years of age (n = 312), the average age was 3.98 ± 1.07 years old (mean ± SD), while for children at 7.7 years old (n = 274), the average age was 7.71 ± 0.72 years. At the age of 7.7 years, 49.3% of the children were boys and 43.2% were defined as white.

The allele and genotype frequencies for the *TAS1R2 Ile191Val* and *TAS1R3 -1266 C/T* polymorphisms are shown in Table 1. The genotype frequency distributions for the variants studied did not differ from those expected for populations under Hardy-Weinberg equilibrium (*TAS1R2 Ile191Val*, χ^2^_GL = 2_ = 0.024, p = 0.988; and *TAS1R3 -1266 C/T* , χ^2^_GL = 2_ = 0.558, p = 0.757). The most frequent allele for *TAS1R2 Ile191Val* was *Ile (72%) and the most frequent allele for *TAS1R3 -1266 C/T* was *C (87%). There were no significant differences in the allele and genotype frequencies between white and non-white children (data not shown).

**Table 1:** Allele and genotype frequencies for the SNPs *TAS1R2 Ile191Val* and *TAS1R3 -1266 C/T*.

SNP	% (n)
*TAS1R2 Ile191Val*	
Genotypes	
*Ile/Ile*	51.4 (160)
*Ile/Val*	40.6 (126)
*Val/Val*	8.0 (25)
Alleles %	
*Ile*	72
*Val*	28
*TAS1R3 -1266 C/T*	
Genotypes	
C/C	74.3 (232)
C/T	24.7 (77)
T/T	1.0 (3)
Alleles %	
C	87
T	13

n = number of carriers of each genotype

After adjustment for confounding variables, 3.9 years old children homozygous (*Val/Val*) for the *TAS1R2 Ile191Val* polymorphism ingested less energy from sugar-dense foods (p = 0.027) and sugars (p = 0.025) than *Ile/Ile* and *Ile/Val* children (Table 2). The percentage variation in BMI from 1 to 3.9 years was not significant among genotypes with the *TAS1R2 Ile191Val* polymorphism, but there was a trend towards a smaller reduction in BMI in *Val/Val* homozygotes (-6.14 ± 9.13%) than in *Ile allele carriers (-9.69 ± 8.83% and -9.82 ± 8.97%; p = 0.052) in this period (Table 2). For 1 and 7.7 years old children, no association was detected between food intake and nutritional status for this variant, but at 7.7 years there was a tendency for *Val/Val* homozygotes to have a greater BMI z-score; there was also greater variation in BMI in 3.9 to 7.7 years old children than in the corresponding *Ile allele carriers. The polymorphism *TAS1R3 -1266 C/T* was not associated with food intake and nutritional status at the three evaluations done in this study.

**Table 2:** Food intake and nutritional status in relation to the *TAS1R2* and *TAS1R3* polymorphisms in Brazilian children.

Food intake and nutritional status	*TAS1R2 Ile191Val*					*TAS1R3 -1266 C/T*		
**1 year old**	*Ile/Ile* (160)	*Ile/Val* (126)	*Val/Val* (25)	P^a^	P^b^	C/C (232)	C/T plus T/T (80)	P
Average daily energy intake (kcal/day)	966.5 ± 395.10	904.4 ± 383.61	867.32 ± 386.17	0.277	0.242	924.8 ± 380.31	970.2 ± 421.0	0.422
BMI Z-score	0.65 ± 1.10	0.62 ± 1.05	0.28 ± 1.18	0.288	0.122	0.59 ± 1.10	0.69 ± 1.04	0.440
**3.9 years old**	*Ile/Ile* (160)	*Ile/Val* (126)	*Val/Val* (25)			C/C (232)	C/T plus T/T (80)	
Average daily energy intake (kcal/day)	1503.5 ± 386.72	1549.6 ± 420.38	1420.0 ± 331.35	0.232	0.233	1528.3 ± 400.23	1490.9 ± 392.41	0.391
SDF (kcal/day)^c^	109.5 ± 84.63	114.7 ± 93.3	78.9 ± 59.6	0.084	0.027	111.2 ± 88.01	103.3 ± 88.83	0.771
Sugar intake (kcal/day)^c^	64.7 ± 63.97	64.4 ± 67.67	44.6 ± 49.1	0.080	0.025	63.3 ± 66.14	63.8 ± 62.7	0.692
BMI Z-score	0.27 ± 1.12	0.22 ± 1.13	0.34 ± 1.05	0.800	0.638	0.21 ± 1.03	0.42 ± 1.34	0.118
BMI variation 1 to 3.9 years (%)	-9.69 ± 8.83	-9.82 ± 8.97	-6.14 ± 9.13	0.148	0.052	-9.67 ± 8.77	-9.04 ± 9.49	0.528
**7.9 years old**	*Ile/Ile* (149)	*Ile/Val* (103)	*Val/Val* (22)			C/C (203)	C/T plus T/T (71)	
Average daily energy intake (kcal/day)	1544.3 ± 406.82	1593.5 ± 337.08	1510.8 ± 335.47	0.286	0.438	1563.8 ± 398.03	1561.0 ± 319.73	0.596
SDF (kcal/day)^c^	80.5 ± 66.67	86.5 ± 86.74	86.5 ± 71.44	0.634	0.510	82.3 ± 75.48	88.35 ± 75.84	0.472
BMI Z-score	0.39 ± 1.44	0.25 ± 1.32	0.88 ± 1.21	0.137	0.068	0.36 ± 1.37	0.46 ± 1.41	0.492
BMI variation 3.9 to 7.9 years (%)^c^	5.87 ± 12.37	4.26 ± 9.62	10.16 ± 13.14	0.116	0.065	5.83 ± 11.83	4.96 ± 10.86	0.759

Values are the mean ± standard deviation; SDF – sugar-dense food.

a General Linear Model: for average daily energy intake, SDF and sugar intake, the intervention or control group status, sex and exact age were used as covariates. For the BMI Z-score, only the variable intervention or control group status was included in the model. For BMI variations, the exact age difference between the child's evaluations, intervention or control group status and sex were included as covariates.

b General Linear Model: the same covariates described above were included, but the comparisons were done between carriers of the *Ile allele *vs. Val/Val* homozygotes.

c For statistical analyses, the variables were natural logarithmic transformed but the non-transformed values are shown in the table.

## Discussion

This study examined the association between two genetic variants of T1R taste receptors and food intake and anthropometric parameters during childhood. The *Ile191Val* polymorphism of the *TAS1R2* gene was found to be associated with differences in the habitual consumption of SDF and sugar intake in children at the age of 3.9 years. The decrease in SDF and sugar intake in *Val/Val* homozygotes was not significantly associated with anthropometric parameters or with variation in weight gain in this cohort. Additionally, contrary to our initial hypothesis, there was a trend towards a higher BMI z-score at 7.7 years old and greater weight gain at 3.9 and 7.7 years old in *Val/Val* homozygotes when compared with *Ile allele carriers. The lack of association of this polymorphism with anthropometric parameters and the trend detected in the opposite direction may be explained by the observation that greater consumption of sugar and SDF did not increase the mean total daily energy intake in *Val/Val* homozygous children.

The functional significance of the *Ile191Val* polymorphism is not yet known and there is currently no information on this matter in the PolyPhen database (http://genetics.bwh.harvard.edu/pph/data/). This variation is located in the conserved large extracellular domain that is one of the ligand-binding regions of the sweet taste receptor (Liao and Schultz, 2003; Nie *et al*., 2005; Kim *et al*., 2006). Eny *et al*. (2010) hypothesized that the *Ile191Val* variant may not be a causal polymorphism but may instead be in linkage disequilibrium with a causal polymorphism. These authors studied the association between this polymorphism and food intake in young adults and diabetics and noted a lack of association with food intake in lean individuals; however, as also observed in the present study, in overweight individuals (BMI > 25) there was a decrease in total carbohydrates, available carbohydrates and total sugar intake for carriers of the *Val allele compared with *Ile/Ile* homozygous individuals.

In the present study, *-1266 C/T* polymorphism of the *TAS1R3* gene was not associated with food intake and nutritional status in three evaluations of the same children in early childhood. The *TAS1R3* gene encodes the receptor subunit of the sweet and the umami receptors. The T1R2-T1R3 taste receptor responds to sweet-tasting compounds, such as sugars, high-potency sweeteners and some D-amino acids, whereas T1R1-T1R3 heteromers comprise an umami taste receptor that is sensitive to L-amino acids (Nelson *et al*., 2001; Zhao *et al*., 2003; Nie *et al*., 2005). Kim *et al*. (2006) proposed that human populations likely show little variation with respect to umami perception, which is controlled by one major form of the receptor that is optimized for detecting glutamate, while there may be a higher level of variation with respect to sweet perception (Kim *et al*., 2006). Studies associating *TAS1R3* gene polymorphisms with variations in sensitivity to sweet taste in humans are still limited. Fushan *et al*. (2009) evaluated 144 unrelated individuals and observed a 25% reduction in the sensitivity to sweet taste in C/T heterozygous individuals. The results of the latter study are not comparable with ours because we evaluated food intake and nutritional status in children and not the sensitivity to sweetness. We hypothesize that to detect an association between a genetic variant of these taste receptors and the habitual consumption of sugar, this variation needs to have a strong effect on protein functionality or expression. In this context, the approach of Fushan *et al*. (2009) is more sensitive for detecting small genetic effect.

Our findings suggest that genetic variation in one component of the T1R2-T1R3 heterodimer may account for interindividual differences in the consumption of sweet foods, although this association was not reflected in modifications in total energy intake or in anthropometric parameters. Our findings contribute to knowledge in the area of taste genetics and may help to explain the different ability of individuals to alter their dietary intake in response to dietary advice. However, this study has some limitations. Only one polymorphism was evaluated for each gene and these polymorphisms were selected based on previous studies describing their possible association with food intake and sweet sensibility. Another relevant feature was the age of the children. In view of their young age, the children may not have had the freedom to choose their foods and they may instead have been supplied with foods that were palatable to adults. In addition, the participants may have consumed all the available foods equally, regardless of whether they presented a greater sensitivity or not, and our data on food intake do not necessarily reflect the sweet sensitivity and food preferences. Furthermore, the food categories analyzed (SDF and sugar intake) may not accurately reflect the issue of palatability and sweet taste because some foods, such as soda, may have a high concentration of sugar but be perceived as less sweet. However, the association detected between genetic variants of the sweet taste receptor gene and SDF and sugar intake makes biological sense. To the best of our knowledge, no other studies have evaluated the association between genetic variations in the taste receptor genes and food intake and nutritional status at different stages of childhood. The results described here may open new perspectives for researchers and contribute to the study of childhood dietary patterns and nutritional status.
